# Editorial Notes

**Published:** 1900-10

**Authors:** 


### TEXAS MEDICAL JOURNAL.

AUSTIN, TEXAS.

A MONTHLY JOURNAL OF MEDICINE AND SURGERY.

EDITED AND PUBLISHED BY

F. E. DANIEL, M. D., AND S. E. HUDSON, M. D.

Published Monthly at Austin, Texas, by Drs. Daniel and Hudson. Subscription price $1.00 a year in advance.

Eastern Representative: John Guy Monihan, St. Paul Building, 220 Broadway, New York City.

Official organ of the West Texas Medical Association, the Houston District Medical Association, the Austin District Medical Society, the Brazos Valley Medical Association, the Galveston County Medical Society, and several others.

### EDITORIAL NOTES.

Dr. Archibald Church has been recently appointed Professor of Nervous and Mental deseases in Northwestern University Medical School-Chicago Medical College, and head of the Neurological Department.

Dr. John B. Murphy has accepted a Professorship in Surgery and Clinical Surgery in the Northwestern University Medical School-Chicago Medical College. Dr. Murphy has been appointed Surgeon-in-Chief of Mercy Hospital, with the direction of the surgical teaching in that hospital. He will give two clinics each week at the hospital. The hospital now contains 260 beds, with abundance of clinical material. A new amphitheatre with a seating capacity of 300 is in progress of construction.

The Charles Roome Parmele Company, of thirty-six Platt Street, New York, through Mr. Parmele, the president, sent the Journal a check for $100 for the relief fund for the sufferers by the cyclone at Galveston. The check was turned over to Col. Wooldridge, President of the City National Bank, who is chairman of the relief committee of this city. This is a most generous and commendable act, and we feel assured that our readers will appreciate it. The Parmele Company is one of the JOURNAL'S staunchest friends and advertising patrons.

---

ROMANCE IN STRICKEN GALVESTON. Dr. Joseph Gilbert, of this city, was engaged to be married to Miss Daisy Thorn, of Galveston. When the city was almost destroyed by the cyclone and tidal wave on Saturday, September 8th, and all communication, even by wire, was cut off, the Doctor of course could not learn the fate of the young lady. He went down on the first train, and managed to get across the bay and into the city. He found Miss Daisy well and safe, at the Tremont Hotel, where so many persons had taken refuge and found safety. The Lucas Terrace, a row of flats, or tenement houses, a popular boarding place, was destroyed, all except the rooms occupied by Miss Thorne, and she and twenty friends were there sheltered during the catastrophy which wrecked all else around and about. Dr. Gilbert lost no time in being married to the lady, and promptly brought her safely away. The ceremony was performed at the Trement, in the midst of wreck and havoc and suffering. The JOURNAL extends congratulations.

---

THE DOCTORS OF GALVESTON seem to bear a charmed life. In the great disaster that befell the city on the 8th of September, in which many thousand lives were lost, not one of the physicians perished. Some were absent from the city, but the great majority were at home, and they exposed themselves and ran the greatest risks; they went at once, midst wave and water, and falling timbers to the rescue of the injured. The earlier dispatches reported our genial and popular Secretary of the State Medical Association, Dr. H. A. West, as lost while edeavoring to get to the Rosenburg school building to the relief of the wounded, but later news reported him safe and well. The quarantine station was wrecked, and Dr. Mayfield, the quarantine officer, and his son were almost miraculously saved; the part of the building in which they took refuge being the the only room which was not blown away. The position of quarantine officer at Galveston is a most hazardous one; several persons having perished at the station in former years. Dr. Peete, the officer there in 1876, it will be remembered, was drowned as well as some members of his family. It is gratifying to state that the splendid medical college building, as well as the great Sealy Hospital, escaped destruction and even serious damage. The St. Mary's, the Sisters' hospital, was partly wrecked.

